# Wild specimens of sand fly phlebotomine *Lutzomyia evansi*, vector of leishmaniasis, show high abundance of *Methylobacterium* and natural carriage of *Wolbachia* and *Cardinium* types in the midgut microbiome

**DOI:** 10.1038/s41598-019-53769-z

**Published:** 2019-11-28

**Authors:** Rafael J. Vivero, Marcela Villegas-Plazas, Gloria E. Cadavid-Restrepo, Claudia Ximena Moreno Herrera, Sandra I. Uribe, Howard Junca

**Affiliations:** 1 Grupo de Microbiodiversidad y Bioprospección, Laboratorio de Biología Molecular y Celular, Universidad Nacional de Colombia sede Medellín, Street 59 A # 63-20, Medellín, Postal Code 050003 Colombia; 2 PECET (Programa de Estudio y Control de Enfermedades Tropicales), Universidad de Antioquia, Street 62 # 52-59, SIU-Sede de Investigación Universitaria, Laboratory 632, Medellín, Postal Code 050003 Colombia; 3 RG Microbial Ecology: Metabolism, Genomics & Evolution, Div. Ecogenomics & Holobionts, Microbiomas Foundation, LT11A, Chía, Postal Code 250008 Colombia; 4 Grupo de Investigación en Sistemática Molecular, Universidad Nacional de Colombia, sede Medellín, Street 59 A # 63-20, Medellín, Postal Code 050003 Colombia

**Keywords:** Microbiome, Microbiome, Biodiversity, Biodiversity

## Abstract

Phlebotomine sand flies are remarkable vectors of several etiologic agents (virus, bacterial, trypanosomatid *Leishmania*), posing a heavy health burden for human populations mainly located at developing countries. Their intestinal microbiota is involved in a wide range of biological and physiological processes, and could exclude or facilitate such transmission of pathogens. In this study, we investigated the Eubacterial microbiome from digestive tracts of *Lu. evansi* adults structure using 16S rRNA gene sequence amplicon high throughput sequencing (Illumina MiSeq) obtained from digestive tracts of *Lu. evansi* adults. The samples were collected at two locations with high incidence of the disease in humans: peri-urban and forest ecosystems from the department of Sucre, Colombia. 289,068 quality-filtered reads of V4 region of 16S rRNA gene were obtained and clustered into 1,762 operational taxonomic units (OTUs) with 97% similarity. Regarding eubacterial diversity, 14 bacterial phyla and 2 new candidate phyla were found to be consistently associated with the gut microbiome content. Proteobacteria, Firmicutes, and Bacteroidetes were the most abundant phyla in all the samples and the core microbiome was particularly dominated by *Methylobacterium* genus. *Methylobacterium* species, are known to have mutualistic relationships with some plants and are involved in shaping the microbial community in the phyllosphere. As a remarkable feature, OTUs classified as *Wolbachia* spp. were found abundant on peri-urban ecosystem samples, in adult male (OTUs n = 776) and unfed female (OTUs n = 324). Furthermore, our results provide evidence of OTUs classified as *Cardinium* endosymbiont in relative abundance, notably higher with respect to *Wolbachia*. The variation in insect gut microbiota may be determined by the environment as also for the type of feeding. Our findings increase the richness of the microbiota associated with *Lu. evansi*. In this study, OTUs of *Methylobacterium* found in *Lu. evansi* was higher in engorged females, suggesting that there are interactions between microbes from plant sources, blood nutrients and the parasites they transmit during the blood intake.

## Introduction

The current situation of leishmaniasis and difficulties for its control^1^, requires integral explorations of various aspects of the vector and parasite lifestyle, nutrient sources, diversity and interplay between insect, feeding regimes and resources, microbiomes, niches, modes of transmission, urban adaptation and influence of human built environments, among others^2^. One initial step to tackle this issue is to describe the microbial diversity associated with the intestine of insect vectors recognized for the transmission of parasites^3–6^. Such knowledge may contribute to understand and to design new alternative control methods, as the ones currently available are evidently insufficient^7^. In Colombia, there are around 14 species of *Lutzomyia* (*Lu*.) reported as vectors of *Leishmania* sp^8–10^. Only the intestinal microbiota of a wild population of *Lu. longipalpis*, and *Lu. evansi* from caribbean coast have been recently reported in other studies^8,11^.

*Lu. evansi*, is recognized because to the vectorial competence that has to transmit parasites causing visceral leishmaniasis in rural and urban environments of the Caribbean coast of Colombia^11–14^. Its abundance and epidemiological importance also makes it an attractive biological model for studies exploring the influence of intestinal microbiota on the fitness, survival, reproduction, parasite transmission and infection establishment in the mammalian host^15^. Therefore, microbiome-based approaches to control vector transmission of *Leishmania infantum* could provide new alternatives and solutions.

Given the environmental plasticity of *Lu. evansi*, the corresponding intestinal microbial communities of this species may require further characterization to see if a similar complex, shifting and diverse composition is observed or if there is a core microbiome with patterns stably maintained between individual specimens. In this context, microbiome studies of insect vectors largely relied on culture techniques used for microbial growth of species aerobic mainly and use to identify associations symbiotic, pathogenic and vectoring. Nevertheless, this approach addresses a limited proportion of the bacterial representation that can be grown on standard conditions, restricting our current knowledge about the microbial communities in insect guts^3,16,17^.

Bacterial diversity analysis by culture-independent means in wild *Lutzomyia* spp. are still scarce. In America, there are reports for sand flies phlebotomine, considering sequences of the 16S rRNA gene, DGGE and NGS with 16S rDNA amplicons^8,18,19^. Gut bacterial types detected on these species are mainly belonging to *Serratia*, *Enterobacter*, *Acinetobacter*, and *Pseudomonas* genera^8,18,19^, probably representing a partial or modified microbiome diversity of the original wild insect population, as studies referred were made in laboratory colonies.

The bacterial diversity of *Lu. intermedia* sand fly from an endemic area in Brazil using metagenomics sequencing was also studied by Monteiro *et al*. 2016, indicating the presence of genera as *Ochrobactrum* and *Bradyrhizobium* across all the groups^5^. Recently, the bacterial gut composition from *Lu. evansi* using cultured and uncultured approaches was described, being similar to the reported above^11^. *Ochrobactrum*, *Shinella*, *Paenibacillus*, *Lysobacter*, *Microbacterium*, *Streptomyces*, *Bacillus*, *Rummeliibacillus*, *Staphylococcus*, *Brevibacterium*, and *Pantoea* were also reported, including microbiota analyses immature states^11^.

Recently, alternative options to the chemical control of insect vectors as sand flies include advances in insect genomics and transformation technology providing new strategies for the control of insect-borne pathogen transmission and insect pest management^19,20^ such as the genetic modification of insects with genes that block pathogen development. Another strategy consist of suppressing insect populations by releasing either sterile males or males carrying female-specific dominant lethal genes into the environment. Considering that arthropods harbor a rich microbiota, its potential egestion after bites may be a shared mechanism that contributes to severity of vector-borne disease, thus, in-depth knowledge of sand flies gut microbiota could help to design new strategies for disease transmission^21^.

Recent studies have highlighted the capacity of endogenous bacteria to modulate viral and parasitic infections in mosquito (*Ae. Aegypti, An. gambiae*) and sand flies vectors (*Lu. longipalpis, P. papatasi*) by activating their immune responses (Toll pathway) or directly inhibiting pathogen development^22–24^. These microbes may prove effective agents for manipulating the vector competence of malaria and other important human pathogens as *Leishmania*^19,25^.

The midgut of insect vectors also a diverse microbiota that can influence insect’s susceptibility to viruses or parasites, and increase their ability to transmit them a more effective to humans^19^. Understanding the interaction between the vector, its microbiota and transmitted pathogens will provide novel opportunities to impact and limit the transmission of leishmaniasis mainly^20^.

There is a specific practical potential of modulating microbiome content to control insect populations and vector capabilities exemplified by applications based on *Wolbachia* bacterial endosymbiont in *Anopheles* spp. and *Aedes* spp^26^. The use of certain strains is also considered a promising alternative for decreasing population density of *Lutzomyia* species and interfering with the multiplication of parasites and, as a result, *Leishmania* transmission^27–29^. Determining the relationship between *Wolbachia* and the development of parasite resistance in sandflies will also open new avenues for exploring biological control, and will provide insights into the role this bacterium plays in populations with high insecticide pressure. However, this is still an analogy of the concept and focused research is required for the *Lutzomyia* pathobiont (microbiome, parasite and insect) system.

The objective of this study was to determine and compared the bacterial composition and diversity between two wild populations of *Lu. evansi*, a key Leshmaniasis vector, collected from Caribbean region in Colombia (tropical biome with high endemism), using high throughput approaches (Illumina MiSeq) based on 16S rRNA gene sequences. We also emphasize in this study in the detection and relative abundances of symbionts.

## Materials and Methods

### Ethics statement

Sandfly collection was performed in accordance with the parameters of Colombian decree N° 1376. No specific permits were required for this study. Sand flies were collected on private property and permission was received from landowners prior to sampling.

### Processing from digestive tracts of *Lu. evansi* adults

**Figure 1:**
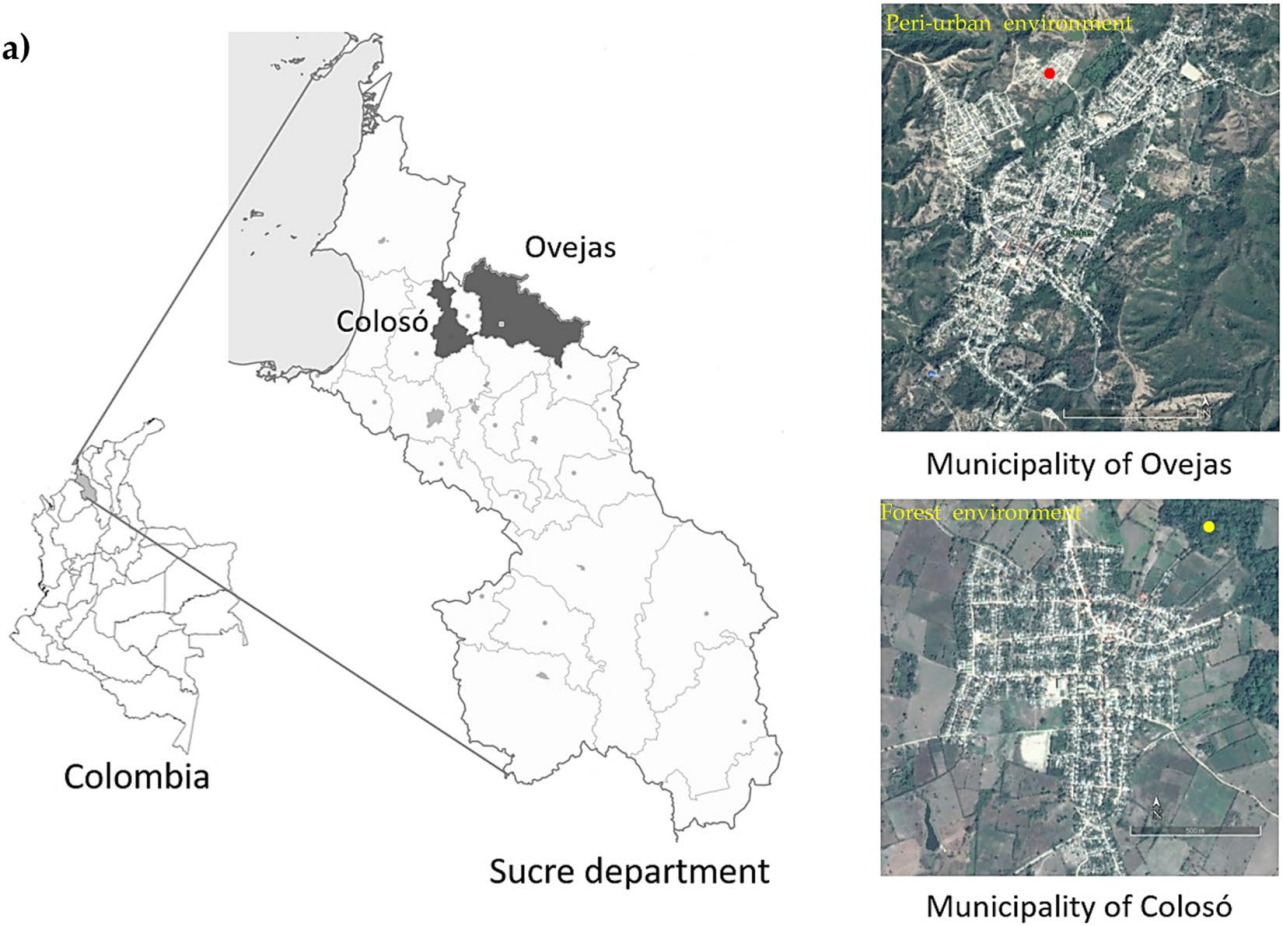
(**a**) Location of the two study areas selected to capture *Lu. evansi* specimens in the department of Sucre, Colombia, Ecoregion Montes de Maria, Caribbean region, Northern Colombia. The red circle symbolizes the collection site in the municipality of Ovejas (Peri-urban environment). The yellow circle symbolizes the collection site in the municipality of Coloso (Forest environment). Prepared by ESRI, U.S. Geological Survey, USGS, National Oceanic and Atmospheric Administration, NOAA. Sundry Civil Bill (U.S. Statutes at Large, v. 20, p. 394–395), 1879.

Sandflies were collected from two locations in the department of Sucre (Caribbean coast of Colombia)^11^. The first location was associated with a peri-urban biotype near to Ovejas municipality (75°13′W, 9°31′N; 277 msnm) (Fig. 1a). The second location corresponded to a forest biotype at the “Primates” Wildlife Experimental Station (09°31′48.0′′N–75°21′4.3′′ W, 220 m) in the municipality of Coloso, located within the “Serranía de Coraza” (Fig. 1a)^11^. The two locations with high incidence of the disease in humans^11^.

Adult specimens were collected from the two locations using Shannon-type extra-domiciliary white light traps that remained active between 18:00 h and 22:00 h^11^. Adult sand flies collected were transported live to the laboratory in entomological cages to obtain gut and morphological structures (male genitalia and female spermathecae) for taxonomic identification of *Lu. evansi* using a taxonomic key^11^. Guts of adult *Lu. evansi* specimens were removed aseptically with sterile stilettos under a stereoscope in 1X PBS buffer^11^. Gut pools were grouped according to locality and physiological stage; these were re-suspended in 100 μL of sterile PBS, before extracting total DNA^11^. The conformation of groups assessed (source, number of intestines, sex, feeding status) and DNA concentrations obtained for sequencing is illustrated in supplementary online material (Table SP1). Samples from each municipality were collected in one single sampling campaign^11^. The number of guts pooled on every group had on average 17.45 ± 2.98 specimens, a retrieval depending of availability in the natural populations at the time of sampling^11^.

### Bacterial 16S rRNA gene fragment PCR amplification and sequencing

PCR amplicon libraries of V4 region of 16S rRNA genes were prepared. Metagenomic DNA was extracted of gut pools from *Lu. evansi* males (Colosó = COM; Ovejas = OVM), fed females (Colosó = COFF; Ovejas = OVFF), and unfed females (Colosó = COUF; Ovejas = OVUF) from both locations^30^. Total gut DNA of *Lu. evansi* was extracted using the Ultra CleanTM Soil DNA Isolation Kit (MO BIO Laboratories, Inc., USA), according to manufacturer’s instructions^30^. Final DNA aliquots were quantified using an ND-100 Nanodrop Thermo Scientific spectrophotometer (Thermo Fisher Scientific Inc, MA). The quality of genomic DNA was analyzed on 1% agarose gel, followed by EZ-visionTM DNA 6X STAIN (AMRESCO)^30^.

Amplicons were obtained using extracted metagenomics DNA as templates on independent reaction where primers 515F [5′-GTGCCAGCMGCCGCGGTAA-3′] and 806R [5′-GGACTACHVGGGTWTCTAAT-3′] were used^30,31^. Each 25 µL PCR reaction comprised 1X buffer, 0.3 mM dNTPs, 0.5 µM forward primer, 0.5 µM reverse primer, 0.04 U/µL Accuzyme polymerase from Bioline and 1 ng of template DNA. PCR cycle conditions were as follows: initial denaturation at 94 °C for 3 minutes followed by 35 cycles of denaturation at 94° for 45 seconds, primer annealing at 50 °C for 60 seconds and extension at 72 °C for 90 seconds^30^. These cycles were finally followed by one last extension at 72 °C for 10 minutes^31^. Sequencing was made using MiSeq Illumina platform and was performed through the Microbiomas Foundation^30^.

### Intestinal microbiome analysis and statistics

All sequence analyses were performed using Quantitative Insight software package into Microbial Ecology (QIIME 1.9.0 version)^32^ and Phyloseq R package 1.5.21^33^. Initially, the complete set of Illumina reads was filtered and split according to barcodes for each sample, chimeric sequences were identified, extracted and excluded out of datasets by Usearch 6.1. A multi-step open-reference OTU picking workflow was performed within QIIME system. Centroid sequence was extracted as a representative sequence for each OTU picked and aligned to SILVA 128 release. In the next steps, a single representative sequence of each OTU was again realigned using PyNAST to build a phylogenetic tree using FastTree^30^. Sequences were deposited in NCBI sequence reads archive (SubmissionID: SUB4855063; BioProject accession: PRJNA507409; BioSample accession: SAMN10492589; Accession: KCPM01000000). All OTUs classified as ascribed to ribosomal sequences from Archaea or from Eukaryotic mitochondria or plastids present and amplified in metagenomic DNA were eliminated and not used in subsequent analyses, as the purpose of this study was to focus on the composition of Eubacterial microbiome^30^.

To estimate species richness and diversity across communities, OTU based analysis was used and implemented using both software package QIIME and Phyloseq R package^32,33^. Based on the OTU assignment, this QIIME workflow script computed measurements of alpha diversity and generated rarefaction plots. Phyloseq R package was used to produce graphics representing abundances, diversity (including Shannon diversity index, observed species, chao1 and Simpson) and distribution of all phyla throughout each sample. Alpha diversity (Shannon_H) and Evenness (Simpson_1-D; Dominance_D; Berger-Parker; Equitability_J) metrics also were estimated in the PAST package, using OTUs associated with core microbiome, with the aim of obtaining a more specific analysis^30^.

Diversity between all the communities (beta diversity) was analyzed by generating Unifrac calculations in both methodologies weighted (presence/absence/abundance matrix) and unweighted (presence/absence matrix) version^30^. This parameter, besides measuring differences between two collections of sequences as the amount of evolutionary history unique to either one, accounts for differences in relative abundances^34^. Using Phyloseq, additional dendrograms were produced out of these calculations. These plots also allowed comparisons of differences and similarities between communities among all the samples^30^. Bray–Curtis dissimilarity matrix was calculated and used it to build Principal Coordinate Analyses and Constrained Principal Coordinate Analysis constrained using Phyloseq R package for your graphically visualized. Differences in the beta diversity of bacterial communities were verified using the non-parametric Permutational Multivariate Analysis of Variance (PERMANOVA) and nonparametric adonis tests, both retrieved from Phyloseq^30^.

Data were analyzed by one-way ANOVA for tested differences in these indices of alpha diversity between groups of guts established, followed by Levene’s Test for Homogeneity of Variance, Shapiro-Wilk test for checking normality, and Durbin Watson test for checking independence^30^. A principal component analysis (PCA) was performed using the “XLSTAT-Ecology” package to identify patterns of bacterial taxa distribution between guts at different stages, sex, and among biotypes^30^.

## Results

**Figure 2:**
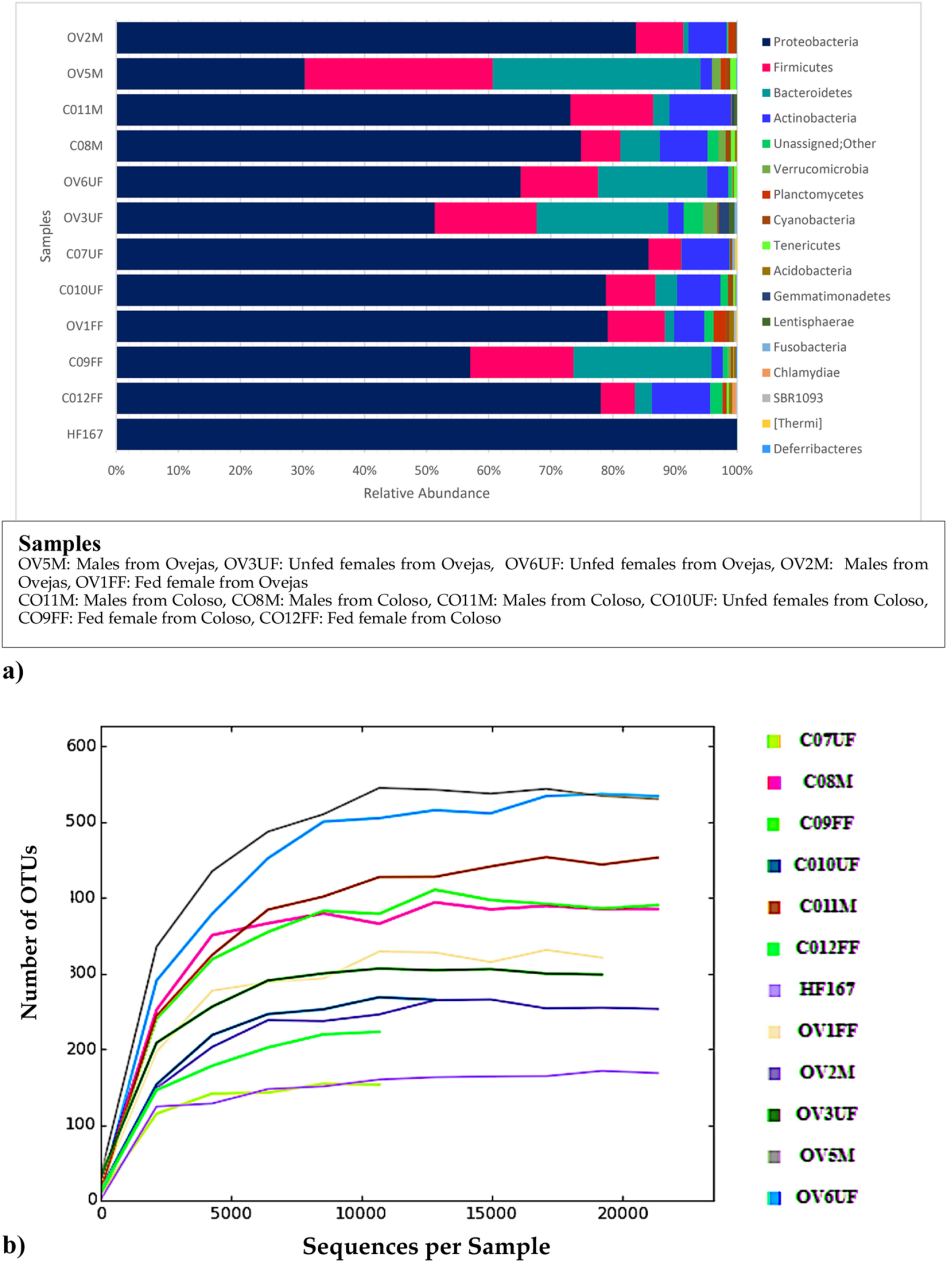
Gut microbiome composition in *Lu. evansi* wild specimens analyzed collected in the Colombian Caribbean region. (**a**) Phyla composition from 16S amplicon Illumina reads on DNA extracted from gut microbiomes. 21.332 sequences per specimen were obtained on average. (**b**) Rarefaction curve from Chao1 analysis using partial 16S rRNA gene sequences of guts from field-collected *Lu. evansi* (male/fed females/unfed females). Saturated rarefaction curve indicates that the vastness of microbial diversity was retrieved from each sample. Nomenclature of gut pools: COM: males from Colosó; COFF: fed females from Coloso; COUF: Unfed females from Coloso; OVM: males from Ovejas; OVFF: fed females from Ovejas; OVUF: unfed females from Ovejas.

335,437 sequences of 250 nucleotides on average were obtained by the Illumina-MiSeq platform, covering region V4 of the 16S rRNA gene. Following the quality filter steps and removal of chimeric sequences, 289,068 reads were clustered into 1,762 OTUs at 97% similarity. Regarding Eubacterial diversity, OTUs recovered from all *Lu. evansi* samples were affiliated with 14 described bacteria phyla and 2 candidate phyla (Fig. 2a).

**Figure 3:**
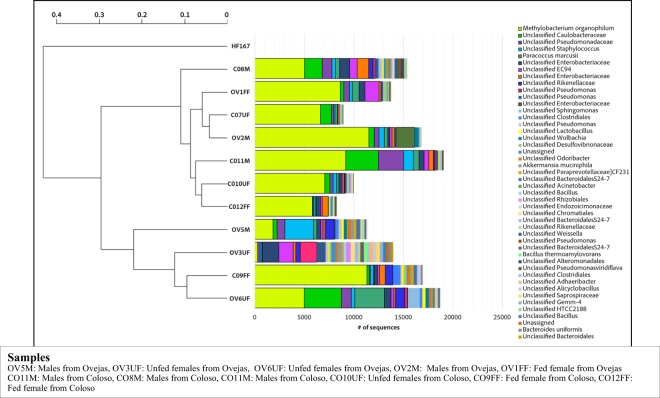
Relative abundance of the set of abundant OTUs with a number of observations equal to or greater than 1% of the total number of sequences analyzed on each sample and weighted UniFrac (left side) dendrogram showing similarities of these community composition. Nomenclature of gut pools: COM: males from Colosó; COFF: fed females from Coloso; COUF: Unfed females from Coloso; OVM: males from Ovejas; OVFF: fed females from Ovejas; OVUF: unfed females from Ovejas.

A rarefaction analysis by Chao1 estimator values revealed a good coverage of sequencing, suggesting that most of the members of all the guts associated community have a description near complete (Fig. 2b). Samples CO10UF, CO7UF and CO9FF were not reaching in this survey a coverage close to saturation; thus, they may still harbor a higher richness of non-detected OTUs. Through this analysis it was possible to determine significant differences in the richness between all samples, being OV5M and OV6UF those with the highest number of OTUs, doubling the number contained of almost all other samples (Figs 2a, 3).

Proteobacteria, Firmicutes and Bacteroidetes were the most abundant phyla in *Lu. evansi*. Proteobateria composed more than 65% of communities in most of the samples, with the exception of OV5M (30%) and OV3UF (50%), which also have the largest fraction of predominant OTUs (Figs 2a, 3). As a proxy to establish a comparable set of predominant OTUs in the communities associated to all specimens, we defined “abundant OTU” as the one with a number of sequences equal to or greater than 1% of the total number of sequences analyzed in each sample (Fig. 3). The resulted in a set of 46 OTUs classified inside five dominant phyla including Proteobacteria, Firmicutes, Bacteroidetes, Verrucomicrobia and Gemmatimonadetes (Fig. 2).

Distances between communities were measured using (Fig. SP1) unweighted UniFrac method to see the difference based on lineages they contain but also on the sum of their counts. Comparison of weighted and unweighted (Figure SP1, Fig. 3) Unifrac analysis supported what can be inferred by the Chao1 estimator; communities from samples OV5M, OV6UF and CO9FF have a significantly higher number of OTUs, but those predominant, have a very lower frequency in that group than in the communities associated with the other specimens. Dendrograms do not show clustering by gender, origin or feeding status, suggesting that these features are not strictly determining the microbiome structure in the set of samples analyzed.

**Figure 4:**
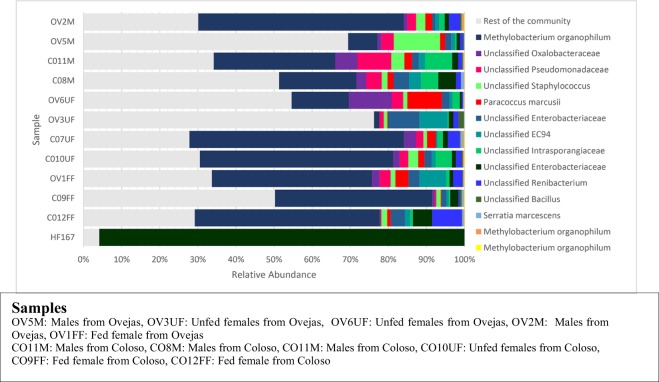
Relative abundance of the set of OTUs comprising the core community and their taxonomical affiliations at the highest resolution obtained (to genera level). Nomenclature of gut pools: COM: males from Colosó; COFF: fed females from Coloso; COUF: Unfed females from Coloso; OVM: males from Ovejas; OVFF: fed females from Ovejas; OVUF: unfed females from Ovejas.

Analyzing the set of shared OTUs between all communities, we defined the core community as the set of OTUs present in all specimens and collectively comprising a major fraction of the communities: We found 14 OTUs with these features that comprised a fraction between 30 to 70% in all communities, most of them belong to the Proteobacteria phylum. The detailed structure and taxonomy composition of core community are presented in Fig. 4. Three different OTUs from *Methylobacterium organophilum* are present in this set and one of them it is also the most abundant one (Fig. 4). OTUs from the families Oxalobacteraceae, Pseudomonadaceae, Infraporangiaceae and Staphylococcaceae are close in abundance within the core community (Fig. 4).

Frequency patterns of the most abundant OTUs revealed that there are three samples; OV6UF, OV5M and OV3UF with a greater number of predominant OTUs, wherein each one comprised a maximum 15% of the community. In all samples, the most abundant OTU was *Methylobacterium organophilum* (Fig. 4), ranging between 30 to 56% in all communities except for the three previously mentioned, where it did not exceed 15%.

**Figure 5:**
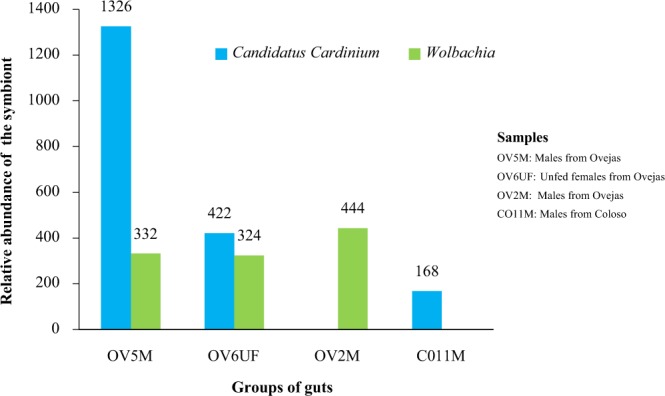
Relative abundance of *Candidatus cardinium* and *Wolbachia* in natural populations of *Lu. evansi* from Sucre. OV5M: males from Ovejas; OV6UF: unfed females from Ovejas; OV2M: males from Ovejas; CO11M: males from Coloso.

A conclusive finding is the detection of OTUs of *Wolbachia* and *Cardinium* endosymbionts in guts from natural populations of *Lu. evansi* (Fig. 5). A high relative abundance of OTUs of *Cardinium* was found mainly in males (OV5M OTUs = 1326) (Fig. 5), and these are related to samples from the Peri-urban (Ovejas) and forest (Coloso) ecosystems (Fig. 5), whereas OTUs of the endosymbiont *Wolbachia* were only identified in the Peri-urban environment and in the intestines of males and females (Fig. 5), in lower relative abundance with respect to *Cardinium* (Fig. 5). Both symbionts are present in unfed females.

**Figure 6:**
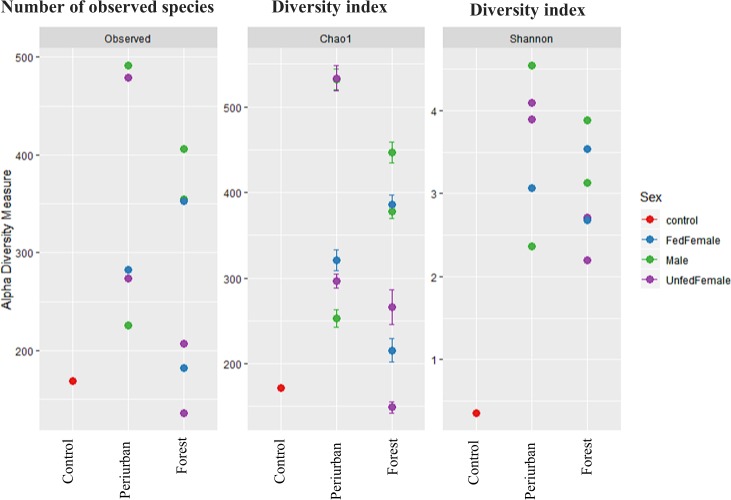
Alpha diversity estimations of gut microbiomes from *Lu. evansi* (male/fed females/unfed females) specimens with respect to environmental habitat, sex and diet. Richness of observed OTUs and Chao1 indexes, and Shannon abundance and evenness indexes. Statistically significant differences in alpha diversity indices were not found (one-way ANOVA Observed df = 2, F = 0.627, P < 0.558; Shannon df = 2, F = 0.235, P < 0.796). Nomenclature of gut pools: COM: males from Colosó; COFF: fed females from Coloso; COUF: Unfed females from Coloso; OVM: males from Ovejas; OVFF: fed females from Ovejas; OVUF: unfed females from Ovejas.

According to the ANOVA, there are no statistically significant differences between groups of guts established from *Lu. evansi* according to the sex or nutritional status (one-way ANOVA, df = 2, F = 0.235, P < 0.796). A greater specific richness is distinguished in the peri-urban ecosystem with respect to the forest ecosystem (Fig. 6). The same behavior is related to Chao1 and Shannon diversity indices. The specific richness is higher in males with respect to females. Only the Chao1 index in unfed females is similar (Fig. 6).

Analysis of alpha diversity of the core gut microbiome of *Lu. evansi* was performed. In terms of species richness, which is directly associated with the number of OTUs per sample, a high number of OTUs was detected in fed females of the two biotypes (OTUs CO9FF = 13655, OTUs OV1FF = 13623) (Table- SP2), as well as in males (OTUs C011M = 18941; OTUs OV2M = 14909). Dominance revealed a higher index in the C09FF sample, which reflects the relative importance of given OTUs (as *Methylobacterium*) or a group (Table S1, Fig. 3), in this case, showing the influence of blood intake in *Lu. evansi* midgut. Simpson diversity index is higher in males of the two biotypes (CO11M, C08M, OV5M) and in unfed females of the peri-urban biotype (Table S1), a greater diversity and changes in shared common species, possibly triggered by the exposure to several microorganism sources. Among the intestine samples of Lu. evansi, we observed a higher uniformity (e ^^^ H/S = 0.5104) of bacterial OTUs in the C08M group of males (Fig. 4).

In all the samples, the equity index does not have values close to 1, suggesting that bacterial OTUs have the same or similar abundances, reflecting highly dynamic bacterial communities within *Lu. evansi* intestines. Berger-Parker index estimated a decrease in equity and an increase in dominance, which is congruent to the detection of a core gut microbiome of *Lu. evansi*. Fed females mainly from the forest environment represent Berger-Parker indices of 0.6853 to 0.8295 (Table S1), so they can have members of some OTUs with a representation of more than half of the individuals, being close to the value of 1.

**Figure 7:**
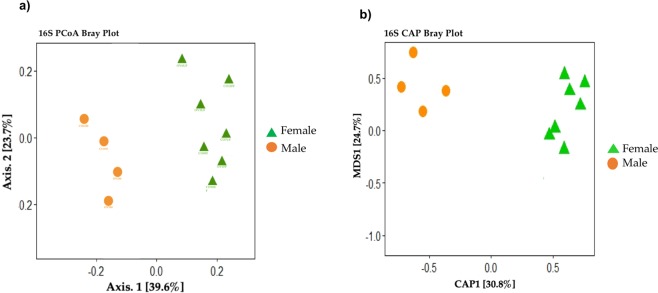
β-diversity analysis of microbial communities associated with the groups of guts established of *Lu. evansi*. (**a**) Constrained Analysis of Principal Coordinates (CAP) of 16S rRNA data (**b**) Principal Coordinate Analysis (PCoA) of Bray-Curtis dissimilarities of 16S rRNA data. Statistical significance of the CAP and PCoA was assessed by Permanova (P < 0.005).

The CAP and PcoA plot (Beta-diversity analysis) show two clustering patterns using Bray–Curtis dissimilarity matrix, which indicate clear differences in the microbiota of males and females mainly (Fig. 7). The statistical analysis (permutation multivariate analysis PERMANOVA and Adonis test) totally reflected PCoA plots results, indicating a significant divergence between groups for both of sex (P = 0.002; R2 = 0.45). The R2 values indicate that ∼45% of the variation in distances can be explained by this grouping.

**Figure 8:**
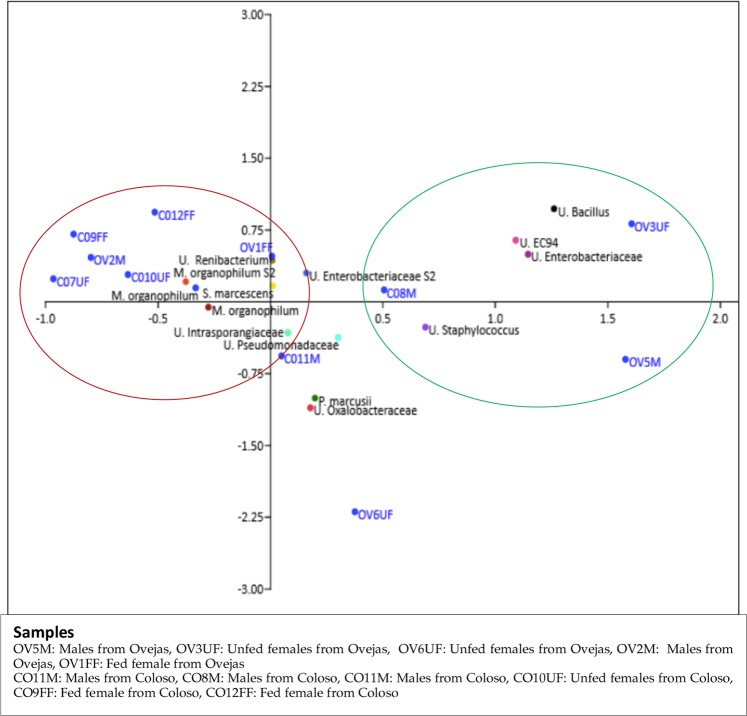
Principal Component Analysis (PCA) showing the distribution of the core microbiome (B) associated with the groups of guts established of *Lu. evansi*. The PCA revealed mainly a cluster matching with fed and unfed females of both locations. Also revealed a cluster with samples of males and unfed. Nomenclature of gut pools: COM: males from Colosó; COFF: fed females from Coloso; COUF: Unfed females from Coloso; OVM: males from Ovejas; OVFF: fed females from Ovejas; OVUF: unfed females from Ovejas.

The principal components analysis (PCA) showed the composing the core microbiome (Figs 4, 8). Fed and unfed females of *Lu. evansi* from peri-urban and forest biotypes has closer associations mainly with *S. marcenscens*, *M. organiphilum* (Three different OTUs), Unclassified *renibacterium*, Unclassified *intrasporangiaceae*. Unlike the samples of *Lu. evansi* appearing closely related to OTUs of Unclassified *Bacillus*, Unclassified EC94, Unclassified Pseudomonadaceae y Unclassified Enterobacteriaceae (Fig. 8).

## Discussion

*Lu. evansi* is an important vector insect in the transmission cycle of visceral leishmaniasis in the Caribbean coast of Colombia, its control is a health priority. For this reason, a complete analysis of their intestinal bacterial microbiome was developed in this work, in order to find possible alternatives for its biological control. A detailed understanding about how pathogens interact with their vectors and the resident microbes can lead to the discovery of new tools to block disease transmission^35^. The first and crucial step in this approach is the identification of suitable commensal bacteria in the vector.

Previous reports of gut microbiome in female sandflies from South America have been performed applying classic techniques of molecular ecology such as DGGE and band sequencing of 16S rRNA gene fragments, and some bacterial types (*Enterobacter*, *Bacillus*, *Serratia*) are usually found in plants and soils, including some sequences of potential plant pathogens or species with entomopathogenic potential^8,11,35^. In this study, we investigated the Eubacterial microbiome from digestive tracts of *Lu. evansi* adults structure using 16S rRNA gene sequence amplicon high throughput sequencing (Illumina MiSeq).

Proteobacteria, Firmicutes, Actinobacteria and Bacteroidetes were the most abundant phyla in all *Lu. evansi* samples. This result is in good agreement with a previous study on the gut microbiota of 81 insects, showing that Proteobacteria and Firmicutes were the predominant phyla, comprising 57.4% and 21.7% of sequences, respectively^36,37^. It is also consistent with microbiota reported to be associated to different species of *Lutzomyia* and *Phlebotomus* genus, as well of mosquitoes (*Aedes* and *Anopheles*) although, Bacteroidetes and Actinobacteria phylum showed lower abundance with respect to our study^3,5,19,38^.

Gut microbiomes on *Lu. evansi* clearly show such abundant phyla shared across all the samples, but also a large fraction of phyla with quite distinct and variable composition even between similar sample groups. The dynamic variation in insect gut microbiota could be explained due to the influence of microhabitats, gut morphology and physicochemical conditions, such as pH, oxygen availability in the insect gut, as well as type of diet^39^. These diverse gut conditions may cause variation in host-specific gut microbiota in insects, influencing nutritional and immune system status as well as reproductive fitness^15^.

Regarding the presence of constant OTUs shared among all *Lu. evansi* samples, notably most dominant *Methylobacterium*, a common cosmopolitan inhabitant of phyllospheres of plants (endophytic, transporting and rhizosphere tissues), thus suggesting the constant feeding from plants in specimens collected^40,41^. Recently, the family Methylobacteriaceae was reported with low representation in (gravid and fed females) *Lu. intermedia*, in an endemic area of leishmaniasis in Brazil^5,38^. These findings together allowed us to suggest that Methylobacteriaceae have the capabilities to remain into the midgut during physiological processes carried out during the blood intake, proposal backed up by experimental evidence of the study published by Kelly *et al*., 2017, showing the continued presence or even increase in abundance of Methylobacteriaceae after the sixth day of feeding and infection with *Leishmania infantum* parasites in *Lu. longipalpis*^19^.

The relative abundance of *Methylobacterium* is significant in salivary glands and midgut of mosquitoes infected and non-infected with *P. falciparum*, which suggests that interactions occur between microbes and parasites^42^. The above indicates that *Methylobacterium* can be important in several physiological and metabolic processes in *Lu. evansi*, which suggests that interactions could occur with *Leishmania* parasite. More studies are needed to deepen in these interactions.

Studies on distribution of *Wolbachia* among its arthropod hosts are important both for better understanding why this bacterium is so common^43,44^, as well as for its potential use as a biological control agent. In America, *Wolbachia* infection has been observed previously in *Lu. cruciata*, *Lu. shannoni*, *Lu. vespertilionis*, *Lu. trapidoi*, *Lu. longipalpis*, *Lu. whitmani*, and *Lu*. intermedia^19,23,45^. One of the main objectives of this study was to confirm, in a new set of samples and with a higher resolution experimental approach the natural presence of *Wolbachia* in the microbiome of *Lu. evansi* specimens wild populations collected in Colombia (department of Sucre). *Wolbachia* endosymbiont was detected in adult male and unfed female specimens, distinctly found only in samples coming from Ovejas. A high proportion of *Wolbachia* was determined, which allows to confirm the preliminary results by conventional PCR^46^, supporting the need of further studies to evaluate the natural infection by *Leishmania* and the presence of this symbiont simultaneously.

Recently, in groups of females fed from *Lu. intermedia*, the family Rickettsiaceae was the most prevalent, with 46.7% of the group represented by *Wolbachia*^5^. This group of females midgut microbiomes was also analyzed by Illumina 16S rRNA gene amplicon sequencing. *Wolbachia* is the microbiota genus most enhanced by the presence of blood meal in the midgut^5^. This is in stark contrast with our study of *Lu. evansi* and for Kelly *et al*. 2017 about *Lu. longipalpis*, in which *Wolbachia* spp., were identified only in sucrose-fed flies and not in blood-fed or infected flies^19,46^.

Despite several reports about the distribution of intracellular symbionts that regulate host reproduction, such as *Wolbachia*, there are few studies addressing the broad distribution of other specific symbionts on insect hosts^47,48^. Among these symbionts, we would like to remark the case of *Cardinium*, which is reported for the first time for *Lu. evansi*, and not reported so far in any other insect vector in Colombia. It is showing a variability between similar groups (ex: *Cardinium* present in one group of OVM, but not in the other one). Wild populations of *Lu. longipalpis* from Brazil had been reported as hosts of this bacterial type^37^. ‘*Candidatus Cardinium*’ is a recently described bacterium from the Bacteroidetes group involved in diverse reproduction alterations of its arthropod hosts (including cytoplasmic incompatibility, parthenogenesis, and feminization) similar to *Wolbachia*^49^. Therefore, this finding could have potential and significance for biological control design. *Cardinium* is also vertically transmitted to progeny but rarely show co-speciation with the host. In sap-feeding insects, as phlebotomine sandflies, plant tissues have been proposed as alternative horizontal routes of interspecific transmission, but experimental evidence is limited^50^.

This remarks the potential of these intracellular bacteria to be studied and considered as possible controlling agents^51,52^; and allows setting new platforms and perspectives to further expand the observations to correlate its presence and abundance in a larger sampled population where *Leishmania* content is also defined on each specimen. Given the recent reports by Kelly *et al*. (2017) and Louradour *et al*. (2017), that providing experimental evidence about the requirement of a gut microbiome linked to the survival of *L. infantum* in *Lu. longipalpis* and of *L. major* in *P. duboscqi* sand flies, respectively^19,53^, this kind of conditioning and controlling of parasite hosting, mediated by the insect gut microbiome, could also may be happening in wild populations of *Lu. evansi*. Further efforts are underway to continue exploring these possibilities.

The presence of OTUs of Oxalabacteraceae and *Staphylococcus* in *Lu. evansi*, is also very remarkable, mainly in males and unfed females. Oxalobacteraceae is a family within the order Burkholderiales in the subclass Betaproteobacteria that contains 13 genera. Possibly these OTUs were acquired by *Lu. evansi* during the larval stage and remained in the intestine until adult. These results correlate with the previous finding of *Burkholderia cenocepacia* in larvae^11^.

It is notable that *Staphylococcus* genera has been associated only to the intestinal microbiota of sandflies from Old World (*Phlebotomus argentipes*, *Phlebotomus chiniensis*, *Phlebotomus papatasi*)^6^, but with significant representation of OTUs, which is similar to the results obtained in *Lu. evansi*. *Staphylococcus* has a variable consistency between hosts, and is mainly related to plants and sap suggesting that they are acquired through sugar feeding^39^.

Microbiota can vary between sexes, feeding status, origin, and status of *Leishmania* infection. Sequencing by Illumina MiSeq platform, allowed us to define the composition of the intestinal microbiota of phlebotomines in the wild^54^. The results presented here open up a promising path to continue the studies on these and other vector species of *Leishmania* in aspects such as enzymatic and antimicrobial activities, influence of microbiota on the resistance to insecticides, and especially, the recalcitrant or favorable effects that specific microbiome components may exert on vector competence.

## Supplementary information

Supplementary Info

## Data Availability

All sequence data generated in this study have been deposited in the European Nucleotide Archive with the accession number (SubmissionID: SUB4855063; BioProject accession: PRJNA507409; BioSample accession: SAMN10492589; Accession: KCPM01000000).

## References

[1] Amora S, Bevilaqua C, Feijo F, Alves N, Maciel M (2009). Control de Phlebotomine (Diptera: Psychodidae) Leishmaniasis Vectors. Neotrop. Entomol..

[2] Saldaña M, Hegde S, Hughes G (2017). Microbial control of arthropod-borne disease. Mem Inst Oswaldo Cruz..

[3] Azambuja P, Garcia E, Ratcliffe N (2005). Gut microbiota and parasite transmission by insect vectors. Trends Parasitol..

[4] Molloy, M., Bouladoux, N. & Belkaid, Y. Intestinal microbiota: shaping local and systemic immune responses. *Semin Immunol*. **24**, 58–66, 1016/j.smim.2011.11.008 (2012). 10.1016/j.smim.2011.11.008 PMC3292882 22178452

[5] Monteiro C (2016). Bacterial diversity of the American sand fly Lutzomyia intermedia using high-throughput metagenomic sequencing. Parasites & vectors..

[6] Fraihi W (2017). An integrated overview of the midgut bacterial flora composition of Phlebotomus perniciosus, a vector of zoonotic visceral leishmaniasis in the Western Mediterranean Basin. Plos Neglected Tropical Diseases..

[7] Rangel E, Vilela M (2008). Lutzomyia longipalpis (Diptera, Psychodidae, Phlebotominae) and urbanization of visceral leishmaniasis in Brazil. Cad. Saúde Pública..

[8] Sant’Anna Mauricio R. V., Darby Alistair C., Brazil Reginaldo P., Montoya-Lerma James, Dillon Viv M., Bates Paul A., Dillon Rod J. (2012). Investigation of the Bacterial Communities Associated with Females of Lutzomyia Sand Fly Species from South America. PLoS ONE.

[9] Bates P (2015). Recent advances in phlebotomine sandfly research related to leishmaniasis control. Parasit Vectors..

[10] Vivero R (2017). Composition and distribution of medically important phlebotomines (Diptera: Psychodidae) in the municipalities of Tierralta and Valencia (Córdoba, Colombia). J Vector Borne Dis..

[11] Vivero J, Jaramillo G, Cadavid-Restrepo G, Soto S, Herrera C (2016). Structural differences in gut bacteria communities in developmental stages of natural populations of Lutzomyia evansi from Colombia’s Caribbean coast. Parasites & Vectors..

[12] González C, Cabrera O, Munstermann L, Ferro C (2006). Distribución de los vectores de Leishmania infantum (Kinetoplastida: Trypanosomatidae) en Colombia. Biomédica..

[13] González Camila, León Cielo, Paz Andrea, López Marla, Molina Gisell, Toro Diana, Ortiz Mario, Cordovez Juan Manuel, Atencia María Claudia, Aguilera Germán, Tovar Catalina (2018). Diversity patterns, Leishmania DNA detection, and bloodmeal identification of Phlebotominae sand flies in villages in northern Colombia. PLOS ONE.

[14] Vergara José Gabriel, Verbel-Vergara Daniel, Montesino Ana Milena, Pérez-Doria Alveiro, Bejarano Eduar Elías (2017). Estimation of time detection limit for human cytochrome b in females of Lutzomyia evansi. Biomédica.

[15] Aksoy Serap (2018). Insect Gut Microbiota: Accessories to the Bite. Cell Host & Microbe.

[16] Degnan P, Ochman H (2012). Illumina-based analysis of microbial community diversity. ISME J..

[17] Li, M. *et al*. Diversity of bacteriome associated with Phlebotomus chinensis (Diptera: Psychodidae) sand flies in two wild populations from China. *Scientific Reports*, 10.1038/srep36406 (2016). 10.1038/srep36406 PMC5098245 27819272

[18] McCarthy B, Diambra A, Rivera P (2011). Metagenomic Analysis of taxa associated with Lutzomyia longipalpis, vector of visceral Leishmaniasis, using an unbiased high-throughput approach. PLoS Neglected Tropical Diseases..

[19] Kelly P (2017). The gut microbiome of the vector Lutzomyia longipalpis is essential for survival of Leishmania infantum. MBio..

[20] Raffa, K. *et al*. Symbionts of invasive insects: characterization, ecological roles, and relation to invasive potential and management strategies. Department of Entomology, University of Wisconsin-Madison, Madison, WI 53706. USDA. *Research Forum on Invasive Species*. 61–62 (2008).

[21] Dey R (2018). Gut Microbes Egested during Bites of Infected Sand Flies Augment Severity of Leishmaniasis via Inflammasome-Derived IL-1β. Cell Host &. Microbiome..

[22] Ramirez Jose Luis, Short Sarah M., Bahia Ana C., Saraiva Raul G., Dong Yuemei, Kang Seokyoung, Tripathi Abhai, Mlambo Godfree, Dimopoulos George (2014). Chromobacterium Csp_P Reduces Malaria and Dengue Infection in Vector Mosquitoes and Has Entomopathogenic and In Vitro Anti-pathogen Activities. PLoS Pathogens.

[23] Xi Z, Ramirez J, Dimopoulos G (2008). The Aedes aegypti Toll Pathway Controls Dengue Virus Infection. PLoS Pathog..

[24] Clayton M, Dong Y, Dimopoulos G (2014). The Anopheles innate immune system in the defense against malaria infection. J Innate Immun..

[25] Meister Stephan, Agianian Bogos, Turlure Fanny, Relógio Angela, Morlais Isabelle, Kafatos Fotis C., Christophides George K. (2009). Anopheles gambiae PGRPLC-Mediated Defense against Bacteria Modulates Infections with Malaria Parasites. PLoS Pathogens.

[26] Hoffmann A, Ross P, Rašić G (2015). Wolbachia strains for disease control: ecological and evolutionary considerations. Evol Appl..

[27] Azpurua Jorge, De La Cruz Dianne, Valderama Anayansi, Windsor Donald (2010). Lutzomyia Sand Fly Diversity and Rates of Infection by Wolbachia and an Exotic Leishmania Species on Barro Colorado Island, Panama. PLoS Neglected Tropical Diseases.

[28] Simoes P, Mialdea G, Reiss D, Sagoi F, Charlat S (2011). Wolbachia detection: an assessment of standard PCR Protocols. Molecular Ecology Resources..

[29] DA ROCHA N. O., LAMBERT S. M., DIAS-LIMA A. G., JULIÃO F. S., SOUZA B. M. P. S. (2017). Molecular detection of Wolbachia pipientis in natural populations of sandfly vectors of Leishmania infantum in endemic areas: first detection in Lutzomyia longipalpis. Medical and Veterinary Entomology.

[30] Vivero, R. Characterization of the intestinal microbiota of natural population of Lutzomyia evansi: vector de visceral leishmaniaisis. *Thesis. Faculty of science, National University of Colombia at Medellin*. http://www.bdigital.unal.edu.co/52971/1/92546771.2016.pdf 160pp (2016).

[31] Caporaso G (2012). Ultra-high-throughput microbial community analysis on the Illumina HiSeq and MiSeq platforms. The ISME journal.

[32] Caporaso, G. *et al*. QIIME allows analysis of high-throughput community sequencing data. *Nat Methods*. **7**(5), 335–336, 10.1038/nmeth.f.303-7 335–33 (2010). 10.1038/nmeth.f.303 PMC3156573 20383131

[33] McMurdie J, Holmes S (2013). Phyloseq: an R package for reproducible interactive analysis and graphics of microbiome census data. PloS One..

[34] Lozupone C, Knight R (2005). UniFrac: a new phylogenetic method for comparing microbial communities. Appl. Environ. Microbiol..

[35] Telleria Erich Loza, Martins-da-Silva Andrea, Tempone Antonio Jorge, Traub-Csekö Yara Maria (2018). Leishmania, microbiota and sand fly immunity. Parasitology.

[36] Colman R, Toolson E, Takacs-Vesbach C (2012). Do diet and taxonomy influence insect gut bacterial communities?. Mol. Ecol..

[37] Yun Ji-Hyun, Roh Seong Woon, Whon Tae Woong, Jung Mi-Ja, Kim Min-Soo, Park Doo-Sang, Yoon Changmann, Nam Young-Do, Kim Yun-Ji, Choi Jung-Hye, Kim Joon-Yong, Shin Na-Ri, Kim Sung-Hee, Lee Won-Jae, Bae Jin-Woo (2014). Insect Gut Bacterial Diversity Determined by Environmental Habitat, Diet, Developmental Stage, and Phylogeny of Host. Applied and Environmental Microbiology.

[38] Pires, A. *et al*. Bacterial diversity of wild-caught Lutzomyia longipalpis (a vector of zoonotic visceral leishmaniasis in Brazil) under distinct physiological conditions by metagenomics analysis. *Parasites & Vectors*. **10**, 627: 10.1186/s13071-017-2593-7 (2017). 10.1186/s13071-017-2593-7 PMC5747039 29284535

[39] Engel P, Moran N (2013). The gut microbiota of insects—diversity in structure and function. FEMS Microbiol. Rev..

[40] Holland, A. *et al*. Methylobacterium spp.: phylloplane bacteria involved in cross-talk with the plant host?. *Phyllosphere microbiology*. p.125-135. In S. E. Lindow, E. I. Hecht-Poinar, and V. J. Elliot (eds), APS Press, St. Paul, Minn (2002).

[41] Green P (2006). Methylobacterium. The prokaryotes..

[42] Jiggins F (2017). The spread of Wolbachia through mosquito populations. PLoS Biol..

[43] Lee S, White V, Weeks A, Hoffmann A, Endersby N (2012). High-Throughput PCR Assays To Monitor Wolbachia Infection in the Dengue Mosquito (Aedes aegypti) and Drosophila simulans. Appl. Environ. Microbiol..

[44] Zug Roman, Hammerstein Peter (2012). Still a Host of Hosts for Wolbachia: Analysis of Recent Data Suggests That 40% of Terrestrial Arthropod Species Are Infected. PLoS ONE.

[45] Ono M, Braig H, Munstermann L, Ferro C, O’NeilL S (2001). Wolbachia infections of phlebotomine sand flies (Diptera: Psychodidae). Journal of medical entomology..

[46] Vivero, J., Cadavid-Restrepo, G., Herrera, C. & Soto, S. Molecular detection and identification of Wolbachia in three species of the genus Lutzomyia on the Colombian Caribbean coast. *Parasites & Vectors*, 10.1186/s13071-017-2031-x (2017). 10.1186/s13071-017-2031-x PMC5329942 28241782

[47] Zchori-Fein, E. & Perlman, S. Distribution of the bacterial symbiont Cardinium in arthropods. *Molecular Ecology*, 10.1046/j.1365-294X.2004.02203 (2004). 10.1111/j.1365-294X.2004.02203.x 15189221

[48] Silva-Gonçalves, D. *et al*. Wolbachia introduction into Lutzomyia longipalpis (Diptera: Psychodidae) cell lines and its effects on immune-related gene expression and interaction with Leishmania infantum. *Parasites & Vectors*. 10.1186/s13071-018-3227-4 (2019). 10.1186/s13071-018-3227-4 PMC6332621 30646951

[49] Gonella E (2015). Plant-mediated interspecific horizontal transmission of an intracellular symbiont in insects. Scientific Reports..

[50] Gruwell M, Wu J, Normark B (2009). Diversity and Phylogeny of Cardinium (Bacteroidetes) in Armored Scale Insects (Hemiptera: Diaspididae). Annals of the Entomological Society of America..

[51] Serbus R, Casper-Lindley C, Landmann F, Sullivan W (2008). The genetics and cell biology of Wolbachia-host interactions. Annual review of genetics..

[52] Brelsfoard C, Dobson S (2011). An update on the utility of Wolbachia for controlling insect vectors and disease transmission. AsPac. J. Mol. Biol..

[53] Louradour Isabelle, Monteiro Carolina Cunha, Inbar Ehud, Ghosh Kashinath, Merkhofer Richard, Lawyer Phillip, Paun Andrea, Smelkinson Margery, Secundino Nagila, Lewis Michael, Erram Dinesh, Zurek Ludek, Sacks David (2017). The midgut microbiota plays an essential role in sand fly vector competence forLeishmania major. Cellular Microbiology.

[54] Knight Rob, Vrbanac Alison, Taylor Bryn C., Aksenov Alexander, Callewaert Chris, Debelius Justine, Gonzalez Antonio, Kosciolek Tomasz, McCall Laura-Isobel, McDonald Daniel, Melnik Alexey V., Morton James T., Navas Jose, Quinn Robert A., Sanders Jon G., Swafford Austin D., Thompson Luke R., Tripathi Anupriya, Xu Zhenjiang Z., Zaneveld Jesse R., Zhu Qiyun, Caporaso J. Gregory, Dorrestein Pieter C. (2018). Best practices for analysing microbiomes. Nature Reviews Microbiology.

