# Supplementary material for: Wild specimens of sand fly phlebotomine Lutzomyia evansi, vector of leishmaniasis, show high abundance of Methylobacterium and natural carriage of Wolbachia and Cardinium types in the midgut microbiome
Source: Sci Rep. 2019 Nov 28;9:17746. doi: 10.1038/s41598-019-53769-z (PMC6883041; doi:10.1038/s41598-019-53769-z)
Supplement: Supplementary file 1 — Supplementary Info [file 41598_2019_53769_MOESM1_ESM.pdf]

Wild specimens of sand fly phlebotomine *Lutzomyia evansi*, vector of leishmaniasis, show high abundance of *Methylobacterium* and natural carriage of *Wolbachia* and *Cardinium* types in the midgut microbiome.

Rafael J. Vivero[1,2*], Marcela Villegas-Plazas[3], Gloria E. Cadavid-Restrepo[1], Claudia X. Moreno Herrera[1], Sandra I. Uribe[4], Howard Junca[3*].

Supporting Information

Figure S1. Unweighted Unifrac distances of the microbiome composition in the gut microbiomes assessed by 16S rRNA gene amplicon sequencing.

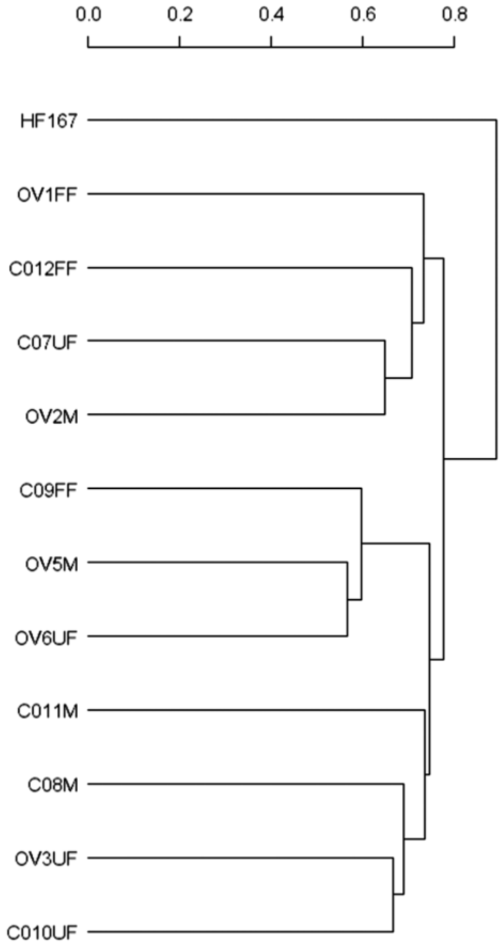

Table S1. Conformation of groups assessed (source, number of intestines, sex, feeding status) and DNA concentrations obtained for sequencing.

Locations-biotype	Sample Code	Sex	Status	Number of guts per group	Total DNA concentration *ng/ul*
Ovejas, Peri-Urban	OV1FF	Female	Fed	20	285
	OV2M	Male	NA	15	435
	OV5M	Male	NA	17	605
	OV3UF	Female	Unfed	20	380
	OV6UF	Female	Unfed	20	380
Coloso, Forest	CO9FF	Female	Fed	20	315
	CO12FF	Female	Fed	16	315
	CO8M	Male	NA	15	340
	CO11M	Male	NA	11	255
	CO7UF	Female	Unfed	18	525
	CO10UF	Female	Unfed	20	285

Table S2. Alpha diversity and evenness metrics associated with the core microbiome of natural populations of *Lu. evansi.* Origin of samples: Males (Colosó=**COM**; Ovejas=**OVM**), fed females (Colosó=**COFF**; Ovejas=**OVFF**) and unfed females (Colosó=**COUF**; Ovejas=**OVUF**).

	**Forest Biotype (Coloso)**						**Peri-Urban Biotype (Ovejas)**				
**Metrics**	**C07UF**	**C010UF**	**C012FF**	**C09FF**	**C011M**	**C08M**	**OV6UF**	**OV3UF**	**OV1FF**	**OV2M**	**OV5M**
**Individuals/OTUs**	8496	9651	8472	13655	18941	12032	15038	4780	13623	14909	7333
**Dominance_D**	0.61	0.54	0.49	0.69	0.27	0.2151	0.22	0.2329	0.4222	0.6032	0.2409
**Simpson_1-D**	0.38	0.45	0.50	0.30	0.72	0.7849	0.78	0.7671	0.5778	0.3968	0.7591
**Shannon_H**	1.01	1.186	1.20	0.84	1.74	1.966	1.783	1.793	1.426	1.061	1.794
**Evenness_e^H/S**	0.19	0.23	0.23	0.16	0.40	0.5104	0.425	0.4291	0.2974	0.2064	0.4293
**Equitability_J**	0.38	0.44	0.45	0.32	0.66	0.7451	0.6757	0.6794	0.5405	0.402	0.6796
**Berger-Parker**	0.77	0.73	0.68	0.82	0.48	0.4165	0.3317	0.346	0.6337	0.7729	0.3967
